# Safety of Immunosuppressive Drugs Used as Maintenance Therapy in Kidney Transplantation: A Systematic Review and Meta-Analysis

**DOI:** 10.3390/ph6101170

**Published:** 2013-09-30

**Authors:** Celline Cardoso Almeida, Micheline Rosa Silveira, Vânia Eloisa de Araújo, Livia Lovato Pires de Lemos, Juliana de Oliveira Costa, Carlos Augusto Lins Reis, Francisco de Assis Acurcio, Maria das Gracas Braga Ceccato

**Affiliations:** 1 Faculdade de Medicina, Universidade Federal de Minas Gerais, Av. Prof. Alfredo Balena, 190, Belo Horizonte/MG, Brazil; 2 Faculdade de Farmácia, Universidade Federal de Minas Gerais, Av. Antônio Carlos, 6627, Campus Pampulha, Belo Horizonte/MG, Brazil; E-Mails: michelinerosa@gmail.com (M.R.S.); vaniaearaujo@gmail.com (V.E.A.); lilolemos@gmail.com (L.L.P.L.); juliana.olic@gmail.com (J.O.C.); carloslinsr@gmail.com (C.A.L.R.); fracurcio@gmail.com (F.A.A.); mgbceccato@gmail.com (M.G.B.C.)

**Keywords:** kidney transplantation, immunosuppressive drugs, adverse events, meta-analysis

## Abstract

To evaluate the safety of regimens containing calcineurin inhibitors (CNI), proliferation signal inhibitors (TOR-I) and antimetabolites, we conducted a meta-analysis of randomized clinical trials (RCTs) and observational studies. A total of 4,960 citations were identified in our electronic search and 14 additional articles were identified through hand searching. Forty-eight articles (11,432 participants) from 42 studies (38 RCTs and four cohorts) met the inclusion criteria. Meta-analysis results revealed the following: (i) tacrolimus was associated with an increased risk for diabetes and lower risk of dyslipidemia, compared to cyclosporine; (ii) mycophenolate mofetil (MMF) was associated with increased risk for total infections, abdominal pain, diarrhea and vomiting, compared with azathioprine; (iii) sirolimus was associated with higher risk of anemia, diabetes, dyslipidemia, lymphoceles and withdrawal compared to tacrolimus or cyclosporine, and cyclosporine was associated with an increased risk of CMV infection; (iv) the combination of CNI with antimetabolites was associated with more adverse events than CNI alone; (v) TOR-I was related to more adverse events than MMF. The data observed in this meta-analysis are similar to those describe by others authors; thus, the choice of treatment must be made by the clinical staff based on specific patient characteristics.

## 1. Introduction

Chronic kidney disease (CKD), most commonly defined as persistent impaired kidney function [1], is a comorbid condition with multiple manifestations that is recognized as an important worldwide public health problem. The prevalence of CKD has increased over the years. It is strongly related with ageing and is more prevalent among women [2,3,4,5] and African Americans [6].

A systematic review of population based studies [7] confirmed that impaired kidney function (iKF) is as common as diabetes mellitus in the general population, and higher quality studies have reported the prevalence of iKF to range from 1.7% in China to 8.1% in the United States. To reduce disease progression, there have been increasing efforts to promote early diagnosis of CKD [8,9]. Without proper care, CKD leads to complications of reduced kidney function, increased risk of cardiovascular disease and, ultimately, kidney failure, the need of renal replacement therapy (RRT) (namely the end-stage renal disease) and death [1].

In terms of RRT, renal transplantation (RT) reduces disability, and improves kidney function and quality of life, and it also provides greater life expectancy and is more cost-effective compared with dialysis [10,11,12]. In 2008, 547,982 United States residents were treated for end-stage renal disease and 17,413 transplants were performed [13].

The effectiveness of immunosuppressive drugs for maintenance therapy has not been a direct issue regarding the success of RT. Nevertheless, choosing the best suitable immunosuppressive therapy is still fairly complex. Multiple classes of drugs are used in combination. Usually, steroids are administrated with calcineurin inhibitors (CNI) such as cyclosporine (CsA) or tacrolimus (TAC) and either proliferation signal inhibitors (TOR-I) such as sirolimus (SRL) and everolimus (EVL) or antimetabolites (AMETAB) such as azathioprine (AZA) or mycophenolate mofetil (MMF) [14].

Moreover, the balance of advantages and disadvantages is used to determine the regimen of choice, because an increasing range of adverse events must be considered when deciding on the optimal immunosuppressive strategy for an individual patient.

New-onset diabetes mellitus (NODAT) is highly associated with CNI treatment, whereas CMV infection is associated with antimetabolites, and dyslipidemia is associated with TOR-I [14,15]. Thus, it is critical to identify and quantify which adverse events are related to a certain drug regimen as this information is missing in the literature.

Therefore, this systematic review and meta-analysis of randomized controlled trials and observational studies was conducted to evaluate the safety of the most commonly used immunosuppressive regimens. The availability of such information would be useful for clinicians when deciding which treatment is most appropriate for each patient.

## 2. Literature Search

This systematic review is reported in accordance with Preferred Reporting Items for Systematic Reviews and Meta-analysis (PRISMA) [16].

### 2.1. Eligibility Criteria and Study Selection

Randomized Controlled Trials (RCTs) and cohort studies comparing treatment regimens that included the immunosuppressants azathioprine, cyclosporine, tacrolimus, mycophenolate mofetil or enteric mycophenolate, sirolimus, or everolimus in any dose and with at least 6 months follow-up were included in this analysis. The eligibility criteria for participants included end-stage renal disease patients over age 16 who had undergone renal transplantation for the first time or not, with a living or deceased donor. Only studies published in English, Portuguese or Spanish were included.

Studies with the following characteristics were excluded: (i) enrolled patients younger than 16 years of age; (ii) did not evaluate RT exclusively; (iii) presented pharmacokinetics or pharmacodynamics results; (iv) were single-arm studies; (v) were non-randomized controlled trials; (vi) were placebo controlled studies; (vii) described results from less than 6 months follow-up; (viii) assessed induction therapy; (ix) were health technology assessments studies; and (x) were studies with a conversion of drugs.

The present study focused on the safety of immunosuppressive drugs; thus, studies that did not report safety information were excluded.

### 2.2. Search Strategy

Several article searches were performed in the Pubmed/MEDLINE, Cochrane Controlled Trials Register, Cochrane’s Renal Group and LILACS databases, covering the period from the inception of the database until August 2013. We also performed a manual search of references that were included in the identified studies and systematic reviews [17,18].

Various combinations of terms were used to search the electronic databases, including terms referring to the disease, interventions and the type of study: “immunosuppression”, “transplant”, “kidney”, “renal”, “azathioprine”, “mycophenolate mofetil”, “cyclosporine”, “tacrolimus”, “sirolimus”, “everolimus”, “effectiveness”, “efficacy” and “safety”.

### 2.3. Selection of Studies and Data Collection

Two independent reviewers performed the study selection in three phases: analyses of titles, abstracts and full-text articles. A third reviewer resolved disagreements regarding eligibility.

After meticulous reading of all included articles, data were extracted in especially designed manual and electronic forms, using Cochrane Review Manager Software—Revman^®^ 5.1 (The Nordic Cochrane Center, Købehvn, Denmark). The studies were classified according to its treatment strategyarms. Comparison between treatment arms was possible if the schemes contained the same concomitant medication (e.g., steroids) at the same dose, differing only by the drug used as intervention and the control.

Any drug-related adverse event and withdrawals were considered safety outcomes. The overall safety outcomes were collected to extract those most prevalent among them. The data were collected in terms of the number of patients who presented a specific outcome. All consolidated data were reviewed to avoid typing errors.

### 2.4. Quality Assessment

The quality of the study was independently accessed by two reviewers, and any disagreements were resolved by consensus. Randomized controlled trials were evaluated using the Cochrane Collaboration Tool [19] considering the following items: random sequence generation, allocation concealment, blinding, and incomplete outcome data. Open label studies were not considered risk of bias. Observational studies were evaluated using Newcastle scale [20].

### 2.5. Data Synthesis and Data Analysis

Outcomes were meta-analyzed if they were reported in at least two articles, within the same treatment arm and at the same time of follow-up. Random effect models were employed to estimate the pooled effect sizes across studies [21]. The results are expressed as the relative risk (RR) with 95% confidence interval (95% CI); *p* value <0.05 was considered significant. To assess heterogeneity I^2^ and *p* values were used (I^2^ >50% and *p* < 0.05 indicated high heterogeneity) Publication bias was accessed using funnel plot. Single analysis (estimated RR from raw data) was performed if the data was not eligible to enter in meta-analysis. All analysis was conducted using Revman 5.1.

## 3. Results

### 3.1. Study Characteristics

**Figure 1:**
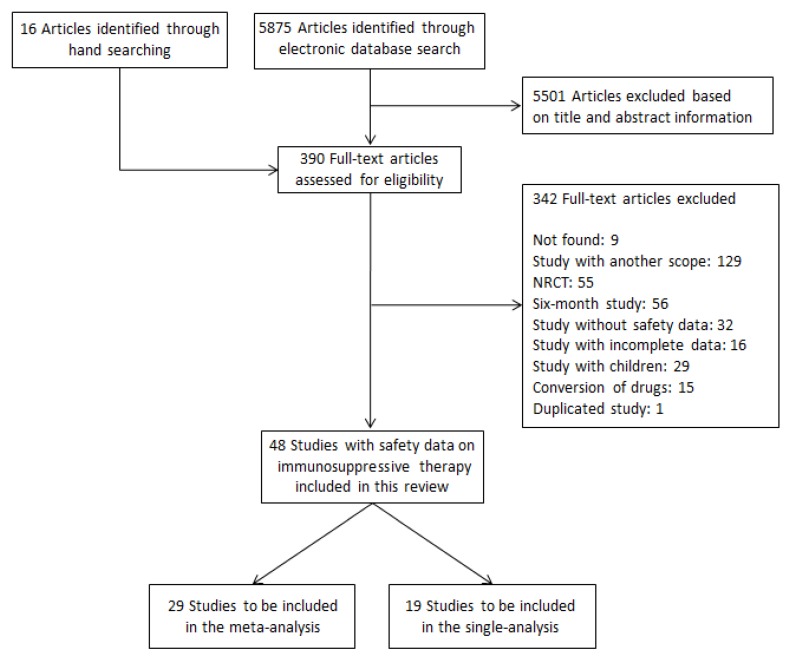
Flow chart of studies included in the systematic review.

A PRISMA flow chart describing the publication screening process and the reasons for exclusion is shown in Figure 1A total of 5,875 citations identified by the electronic search, and 16 additional articles were identified via manual searching. A total of 48 articles (11,432 participants), from 42 studies, 38 RCTs [22,23,24,25,26,27,28,29,30,31,32,33,34,35,36,37,38,39,40,41,42,43,44,45,46,47,48,49,50,51,52,53,54,55,56,57,58,59,60,61,62,63,64,65] and four cohorts [66,67,68,69] met the inclusion criteria.

Table 1 shows the characteristics of included studies organized by treatment strategy, according to the treatment protocol of each study. CsA was the most prevalent drug in all schemes, as it was used in 34 articles. The majority of studies (33%) compared TAC versus (*vs.*) CsA, usually using an antimetabolite and a steroid in combination. Within each study, differences between groups in terms of gender, race, age and allograft characteristics were not significant, indicating that the allocation of participants into the treatment groups was satisfactory. Twenty five RCTs were multicenter studies, with the number of centers ranging from 2 to 65 centers. Nearly 50% of the studies were conducted in European countries, 21% of the studies were conducted in the United States, and 14% were conducted in Brazil.

**Figure 2:**
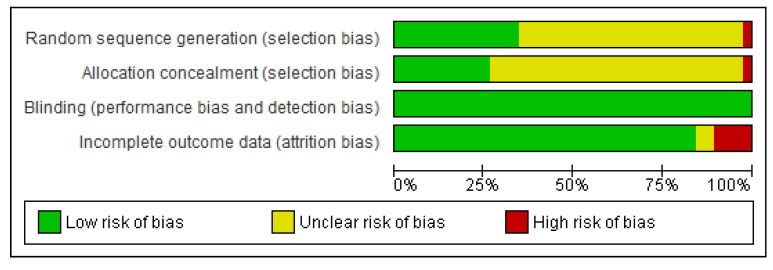
Quality of RCTs included in the review.

Of the 38 RCT studies evaluating immunosuppressants only 13 (34%) reported adequate sequence generation and most studies did not report the allocation concealment (73%). One study [34] used the numbers of records for randomization and was classified as high risk for selection bias. All studies, but four [22,23,26,40] used intention to treat or modified intention to treat analysis. A summary of RCT quality is shown in Figure 2. The four included cohorts [66,67,68,69] assigned three or four stars in the selection domain, one star in the comparability of groups and one to two in the exposure, demonstrating good quality. Funnel plots of meta-analyses were all symmetrical, indicating the absence of bias.

**Table 1:** Characteristics of included studies.

Study (year)	Treatment	N (female %)	White %	First Transplant %	Deceased Donor %	Cold Ischemia time (SD)	Mean Donor Age (SD)	Mean Age (SD)	Study design, location, time of follow-up and funding
**1. CNI *vs.* CNI**									
1.Scantleburry (1991) [22]	CsA + Pred TAC + Pred	20 14	NR NR	100 100	NR NR	NR NR	NR NR	NR NR	RCT, USA, single center, 12 months
2. Mayer (1997) [23]	CsA + AZA + Pred TAC + AZA + Pred	145 (36.6) 303 (35.3)	NR NR	89.7 90.4	100 100	NR NR	43.0 45.2	45.8 46.6	RCT, England, multicenter (15), open label, 12 months, funded by Fujisawa GMBH
3. Yang (1999) [24]	CsA + MMF + Ster TAC + MMF + Ster	30 (37) 30 (50)	87.0 77.0	NR NR	57.0 67.0	15 (1.6) 14 (1.5)	37 (2.6) 39 (3.0)	48.0 (2.2) 45.0 (2.4)	RCT, USA, single center, open label, 12 months
4. Wang (2000) [25]	CsA + MMF + Pred TAC + MMF + Pred	32 25	NR NR	NR NR	100 100	NR NR	NR NR	38.1 (18.7)	RCT, China, single center, 12 months results
5. Nichelle (2002) [26]	CsA + AZA + Ster TAC + AZA + Ster	46 48	NR NR	NR NR	NR NR	NR NR	NR NR	NR NR	RCT, France, single center, 12 and 36 months
6. Campos (2002) [27]	CsA TAC	81 (44) 85 (52)	NR NR	94 96	52.0 46.0	NR NR	37.5(14.3) 36.5(13.7)	40.9 (12.3) 40.5 (10.7)	RCT, Brazil, multicenter (15), open label, 12 months
7. Murphy (2003) [28]	CsA + Pred + AZA TAC + Pred + AZA	50 (30.0) 52 (38.5)	NR NR	88.0 88.0	84.0 82.0	LD:1.7; CAD:19.0; NHBD:15.5; LD: 2.2; CAD:18.7; NHBD:15.1	LD:49; CAD:44; NHBD:48; LD:45; CAD:39; NHBD:49	45.0 (12.0) 45.0 (14.0)	RCT, England, multicenter (2), open label, 12 months
8. Jurewicz (2003) [66]	CsA + AZA + Ster TAC + AZA + Ster	117 115	NR NR	NR NR	NR NR	NR NR	NR NR	NR NR	Cohort, United Kingdom, single center, 72months
9. Hardinger (2005) [29]	CsA + AZA + Ster TAC + AZA + Ster	66 (39) 134 (36)	79 79	100 100	51.0 58.0	12 (4) 13 (5)	NR NR	44,0 (13.0) 46,0 (13.0)	RCT, USA, single center, open label, 12 months
10. Fukuhara (2005) [67]	CsA + Pred TAC + Pred	137 (36.5) 55 (30.9)	NR NR	NR NR	100 100	11.95 (6.12) 12.13 (6.58)	47 (18) 46 (16)	44 (9) 42 (11)	Cohort, Japan, single Center, 10 years
11. Silva (2006) [68]	CsA + AZA/MMF TAC + AZA/MMF	80 (44) 68 (50)	50 53	91 85	100 100	21 (8) 18 (7)	34 (14.0) 34 (12.0)	42 (12.0) 43 (12.0)	Cohort, Brazil, single center, 12 months
12. Silva, Jr. (2007) [30]	CsA + Pred TAC + Pred TAC XL + Pred	212 (35.5) 212 (35.8) 214 (38.7)	76.9 71.7 74.8	95.8 96.3 96.3	47.6 50.0 51.9	18.44 (7.11) 19.41 (7.27) 17.88 (7.73)	NR NR NR	47.6 (13.0) 48.6 (12.9) 47.8 (13.0)	RCT, Brazil, , multicenter (60), open label, 12 months, funded by AstellasPharma US
13. Cheung (2009) [31]	CsA + Pred TAC + Pred	38 (34.2) 38 (44.8)	100^a^ 100^a^	100 100	100 100	8.7 (4.6) 9.1 (5.1)	48.9 (13.2) 48.9 (13.2)	40.2 (11.7) 41.8 (7.5)	RCT, China, single center, open label, 60 months
14. Vicenti (1996) [32]	CsA TAC	28 (21.4) 92 (34.8)	53.6 51.1	100 100	NR NR	NR NR	NR NR	46.6 44.1	RCT, USA, multicenter (5), open label, 12 months
**2. CNI *vs.* CNI + AMETAB**									
1. Moreso (1998) [33]	CsA + Pred CsA + MMF(ld) + Pred CsA + MMF + Pred CsA(ld) + MMF + Pred	27 (48.2) 27 (44.4) 28 (42.9) 15 (33.3)	NR NR NR NR	37.0 44.4 46.4 93.4	100 100 100 100	NR NR NR NR	41 (16) 41 (18) 42 (17) 44 (14)	47 (15) 45 (14) 43 (15) 47 (7)	RCT, Spain, multicenter (2), double blind in the standard dose CsA groups and open label in the low-dose CsA, 24 months
2. Raofi (1999) [34]	CsA + AZA TAC + Pred	21 (27.7) 14 (22.9)	100^b^ 100^b^	100 100	100 100	26 (10) 25 (8)	NR NR	46.0 (11.0) 44.0 (14.0)	RCT, USA, single center, 12 months
3. Sandrini (2000) [35]	CsA + Pred CsA + AZA + Pred	58 (45.0) 58 (38.0)	NR NR	100 100	100 100	NR NR	35 (14) 35 (16)	42 (11) 44 (10)	RCT, Italy, single center, 60 months
4. Segoloni (2000) [36] [Pascual (2003)] [37]	TAC + Pred TAC + AZA + Pred	236 (35.2) 239 (35.6)	NR NR	NR NR	100 100	18.0 17.6	NR NR	46.0 45.0	RCT, Italy and Spain, multicenter (36), open label, 12 and 36 months
5. Chang (2001) [38]	TAC + Ster TAC + AZA + Ster	124 (37.9) 121 (32.2)	77,4 76,0	NR NR	NR NR	20.4 21.3	NR NR	48.0 45.0	RCT, United Kingdom, multicenter (08), open label, 12 months
6. Squiflet (2001) [39]	TAC + Pred TAC + MMF(ld) + Pred TAC + MMF + Pred	82 (46.3) 79 (32.9) 71 (36.6)	93.9 97.5 95.8	86.6 87.3 90.1	100 100 100	NR NR NR	45.6 (18.1) 45.6 (16.0) 45.4 (16.9)	46.6 (14.5) 46.5 (13.3) 48.0 (13.3)	RCT, Belgium, multicenter (16), 12 months, funded by Fujisawa
**3. CNI vs. AMETAB**									
1. Hall (1988) [40]	CsA AZA + Pred	138 (42.8) 138 (45.0)	NR NR	100 100	100 100	22.0 22.7	NR NR	NR NR	RCT, Australia, multicenter (7), 36 months, funded by Sandoz
2. Schnuelle (2001) [41]	CsA + Ster MMF + Ster	44 (27.3) 40 (45.0)	NR NR	95,5 97,5	NR NR	21.7 (9.0) 21.0 (7.5)	40.7 (15.3) 47.7 (15.4)	44.7 (13.3) 51.3 (11.5)	RCT, Germany, multicenter (3), open label,12 months
3. Hamdy (2008) [42]	TAC + SRL + Pred MMF + SRL + Pred	65 (20.0) 67 (29.8)	NR NR	100 100	0 0	NR NR	35.6 (10.3) 36.2 (10.2)	32.3 (10.3) 31.8 (8.6)	RCT, Egypt, single center, 63 months
**4. CNI *vs.* TOR-I**									
1. Groth (1998) [43]	CsA + AZA + Pred SRL + AZA + Pred	42 (40.0) 41 (29.0)	88.0 98.0	100 100	100 100	17.4 (7.2) 18.9 (7.4)	37.7 (15.9) 44.6 (13.4)	41.6 (11.8) 47.5 (10.8)	RCT, Sweden, multicenter (11), open label, 12 months
2. Büchler (2007)[44]Lebranchu (2012) [45]	CsA + MMF + Ster SRL + MMF + Ster	74 (39.2) 71 (38.0)	95.9 94.4	89.2 95.8	100 100	20.17 (5.46) 19.30 (5.24)	41.3 (14.0) 38.7 (14.4)	45.1 (12.4) 45.6 (10.3)	RCT, France, multicenter (13), 12 months, funded by Wyeth
3. Guba (2010) [46]	CsA + MMF + Ster SRL + MMF + Ster	71 70	98.6 98.6	89.9 94.4	88.4 90.1	13.0 (7.0) 12.1 (5.7)	47.1 (14.3) 46.9 (14.3)	47.1 (11.1) 47.0 (10.8)	RCT, Germany, multicenter (9), 12 months, funded by Wyeth and Fresenius Biotech
4.Glotz (2010) [47]	TAC + MMF + Ster SRL + MMF + Ster	70 71	91.4 77.5	94.3 94.4	100 100	18 (6) 19 (5)	45.1 (12.6) 45.2 (13.4)	46.7 (10.6) 48.5 (9.5)	RCT, France, multicenter (13), 12 months, funded by Wyeth
**5. CNI + AMETAB *vs.* CNI + AMETAB vs. CNI + AMETAB**									
1. Hernandez (2007) [48]	CsA + AZA + Ster CsA + MMF + Ster TAC + MMF + Ster	80 (26.2) 80 (37.5) 80 (45.0)	NR NR NR	100 100 100	42 50 59	20.3 (4) 21.0 (4) 21 (4)	45 (16) 42 (15) 44 (17)	47 (12) 48 (14) 47 (11)	RCT, Spain, single center, open label, 24 months, funded by Spanish Health Ministry
**6. AMETAB *vs.* AMETAB**									
1. Keown (1995) [49]	AZA + CsA + Pred MMF + CsA + Pred MMF(hd) + CsA + Pred	173 (46.2) 166 (33.1) 164 (40.2)	NR NR NR	10.4 14.46 10.98	NR NR NR	20 (7) 21 (9) 20 (7)	38 (16) 39 (16) 37 (16)	46 (13) 47 (13) 46 (13)	RCT, Canada, multicenter (21), double blind, 24 months
2. Pescovitz (1998) [50] [Pescovitz (2001)] [51]	AZA + CsA + Ster MMF + CsA + Ster	108 (40.7) 113 (36.3)	68.5 21.3	87 91	NR NR	NR NR	NR NR	43.7 (11.7) 43.1 (11.6)	RCT, USA, multi centric (15), double blind, 12 and 36 months
3. Folkmane (2002) [52]	AZA + CsA + Pred MMF + CsA + Pred	23 23	NR NR	NR NR	100 100	NR NR	NR NR	43.2 (12.1) 43.2 (12.1)	RCT, Lithonia, 12 months
4. Sadek (2002) [53]	AZA + CsA + Pred MMF + CsA + Pred	157 (29.0) 162 (40.1)	91.4 90.4	100 100	87 86	NR NR	NR NR	43.9 (12.8) 43.9 (13.0)	RCT, United Kingdom, multicenter (28), open label, 12 months, funded by Novartis
**7. AMETAB *vs.* TOR-I**									
1. Vitko (2004) [54] [Vitko (2005)] [55]	MMF + CsA EVR(hd) + CsA EVR(ld) + CsA	194 198 196	NR NR NR	100 100 100	NR NR NR			NR NR NR	RCT, Czech Republic, multicenter (54), double blind, 12 and 36 months, funded by Novartis
2. Lorber (2005) [56]	MMF + CsA + Pred EVR(hd) + Csa + Pred EVR(ld) + CsA + Pred	196 (32.7) 194 (36.6) 193 (29.5)	65.8 63.4 70.5	100 100 100	45.9 51.5 52.3	CAD:18.6 (6.42); LD:1.3 (1.16); CAD:18.8 (6.43); LD:1.2 (1.14) CAD:19.5 (7.18); LD: 1.4 (3.4)	36.7 (13.81) 38.4 (13.66) 37.4 (13.55)	43.4 43.7 43.3	RCT, Switzerland, multicenter (44), 36 months, funded by Novartis
3. Mendez (2005) [57]	MMF + TAC + Pred SRL + TAC + Pred	176 (30.1) 185 (33.5)	54.0 50.8	NR NR	64.2 63.2	19.8 19.1	NR NR	47.8 (12.3) 45.3 (12.4)	RCT, USA, multicenter (27),open label, 12 months, funded by Fujisawa
4. Sampaio (2007) [58]	MMF + TAC + Pred SRL+TAC+Pred	50 (24.0) 50 (38.0)	54.0 42.0	100 100	24.0 24.0	NR NR	41.9 (10.5) 41.6 (10.0)	42.6 (14.2) 37.4 (10.3)	RCT, Brazil, single center, open label, 12 months, funded by Janssen-Cilag
5. Tedesco-Silva (2010) [59]; Cibrik (2013) [60]	MMF EVR EVR(ld)	277 (31.8) 279 (31.5) 277 (36.5)	68.6 64.5 69.7	100 100 100	46.2 45.9 46.6	NR NR NR	41.8 (13.6) 41.1 (13.0) 41.4 (13.9)	47.2 (12.7) 45.3 (13.4) 45.7 (12.7)	RCT, Brazil, multicenter, open label, 12 and 24 months, funded by Novartis
**8. CNI *vs.* CNI *vs.* TOR-I**									
1. Ekberg (2007) [61]; Ekberg (2009) [62]	CsA(sd) + MMF + Ster CsA(ld) + MMF + Ster TAC(ld) + MMF + Ster SRL(ld) + MMF + Ster	384 (37.7) 408 (33.6) 403 (34.2) 380 (33.3)	92.1 92.2 94.0 94.2	NR NR NR NR	65.6 64.2 62.8 64.2	16.6 (5.5) 16.8 (5.2) 16.5 (5.7) 16.0 (5.8)	44.6 (15.9) 46.2 (15.1) 45.2 (15.5) 46.0 (14.8)	45.9 (13.8) 47.2 (13.5) 45.4 (14.7) 44.9 (14.5)	RCT (12 months) and Cohort (36 months), Sweden, multicenter (15), open label, 12 and 36 months, funded by Hoffman-La Roche
**9. CNI+AMETAB *vs.* CNI+TOR-I**									
1.Kumar ^†^ (2005) [63]	CsA + MMF CsA + SRL TAC + MMF TAC + SRL	58 52 50 40	AA = 0 N − AA = 89	AA = NR Non – AA = NR	AA = 93 n − AA= 83	AA = 15.5 (6.8) n − AA = 15.9 (12.1)	AA = 42.0 (16.5) N − AA = 42.3 (19.2)	AA = 52.9 (12.0) n − AA = 53.0 (15.6)	RCT, USA, single center, 12 months
**10. TOR-I *vs.* CNI+TOR-I**									
1. Tedesco-Silva (2010) [64]	SRL SRL+CsA	102 (36.3) 105 (36.2)	72.6 62.9	98.0 98.1	31.4 30.5	7.36 (0.99) 7.64 (1.03)	NR NR	41.5 40.9	RCT, Brazil, multicenter (9), open label, 12 months, funded by Wyeth
**11. CNI+AMETAB *vs.* CNI vs. AMETAB**									
1. Gheith (2008) [69]	CsA + AZA + Pred CsA + Pred AZA + Pred	239 (26.36) 75 (42.67) 130 (26.92)	NR NR NR	NR NR NR	0 0 0	NR NR NR	34.0 (9.2) 34.6 (10.3) 33.3 (10.1)	30.7 (10.1) 28.1 (10.3) 29.8 (7.9)	Cohort, Egypt, single Center, 20 years
**12. TOR-I + CNI-Elim *vs.* TOR-I *vs.* CNI**									
1. Flechner (2011) [65]	SRL + TAC-Elim SRL + MMF TAC + MMF	152 (28.3) 152 (27.6) 139 (41.7)	75.0 77.0 73.4	92.8 91.5 92.1	60.5 63.2 64.0	17.7 (6.7) 17.3 (5.7) 17.4 (6.3)	43.2 (13.6) 45.5 (14.9) 44.4 (13.9)	47.9 (13.3) 50.4 (13.0) 48.4 (13.2)	RCT, USA, multicenter (65), open-label, 24 months, funded by Wyeth

Abbreviations: AMETAB, Antimetabolites; CAD, Cadaveric donor; Elim, elimination; LD, Living donor; NHBD, Non-heart beating donor; NR, not reported; Pred, Prednisone; Ster, Steroids; (ld), Low dose; (sd), Standard dose; (hd), High dose. ^†^ The study compares AA (African American) and n-AA (non-African American) recipients

### 3.2. Outcomes

All adverse events reported in the included articles were collected, and the most prevalent events were included in the synthesis. The following outcomes were included: abdominal pain, anemia, bacterial infections (all definitions), cytomegalovirus (CMV) infections, diabetes mellitus (new-onset diabetes mellitus, post-transplant diabetes, and use of hypoglycemic drugs were considered), diarrhea, dyslipidemia (hypercholesterolemia, hypertriglyceridemia and hyperlipidemia were considered), gastritis, total infections (as reported in the study), hypertension (use of antihypertensive drugs was also considered), leukopenia, lymphoceles, malignancies (all types), nausea, vomiting, thrombocytopenia, urinary tract infection (UTI) and withdrawal (discontinuation and crossover of study medication were considered).

For data synthesis and analysis, the comparable schemes in each study were classified into the following groups: CNI *vs.* CNI; AMETAB *vs.* AMETAB;TOR-I *vs*. CNI; CNI + AMETAB *vs*. CNI; TOR-I *vs*. AMETAB; and AMETAB *vs.* CNI. In some studies, it was possible to compare more than one group, such as studies that included the treatment protocol of CNI + AMETAB *vs.* CNI + AMETAB *vs.* CNI + AMETAB (it was possible to compare CNI *vs.* CNI and AMETAB *vs.* AMETAB).

#### 3.2.1. CNI *vs.* CNI

All studies that compared CsA and TAC were included in this group. A total of 17 articles [22,23,24,25,26,27,28,29,30,31,32,48,61,62,66,67,68] reported safety data related to TAC as the experimental treatment and CsA as the control. One study used low-dose TAC (3–7 ng/mL) and low-dose CsA (50–100 ng/mL) [61,62], whereas the others used standard doses of both drugs (5–15 ng/mL for TAC and 150–300 ng/mL for CsA).

**Figure 3:**
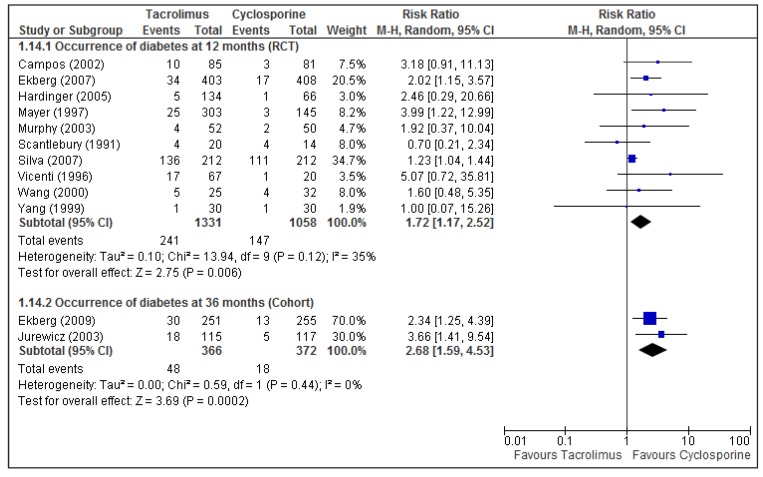
Meta-analysis of diabetes for TAC *vs.* CsA comparison at 12 and 36 months.

The results of 13 articles, two cohorts and 11 RCTs, were meta-analyzed and are displayed in Table 2. Both the cohort and RCT pooled results indicate that TAC was associated with an increased risk for diabetes (Figure 3). This association was also found at 120 months follow-up in one cohort that was not included in the pooled analysis (n = 192; RR = 2.10; 95% CI: 1.17, 3.77; *p* = 0.01) [67]. The risk of dyslipidemia was reduced in TAC regimens, as shown in the meta-analysis and in two single studies: a cohort of 36 months (n = 506; RR = 0.74; 95% CI: 0.57, 0.97; *p* = 0.03) [62] and a RCT of 60 months (n = 76; RR = 0.62; 95% CI: 0.40, 0.95; *p* = 0.03) [31].

Although the other outcomes had no statistical significance in the pooled results, studies in single analysis showed that TAC was associated with a higher risk of withdrawing the treatment at 120 months of follow-up (n = 192; RR = 11.21; 95% CI: 2.50, 50.23; *p* = 0.002) [67] and that CsA presented a greater risk for hypertension at 36 months of follow-up (n = 89; RR = 0.67; 95% CI: 0.48, 0.94; *p* = 0.02) [26].

**Table 2:** Meta-analysis results of outcomes reported by studies comparing TAC *vs.* CsA ^a^.

Outcome	Study Design (N)	Time in months	Relative Risk ^b^ (95% CI)	Statistics ^c^	
				*p*	I^2^
CMV	RCT [23,24,29,61] (1519)	12	0.85 (0.64, 1.15)	0.30	0
Diabetes	RCT [22,23,24,25,27,28,29,30,32,61] (2389)	12	1.72 (1.17, 2.52)	0.006	35
	Cohort [62,66] (738)	36	2.71 (1.61, 4.57)	0.0002	0
Dyslipidemia	RCT [29,30,61] (1435)	12	0.75 (0.60, 0.94)	0.01	0
Hypertension	RCT [23,26,27,29,61] (1714)	12	0.97 (0.82, 1.16)	0.76	25
Total Infections	RCT [23,24,25,61] (1376)	12	1.03 (0.93, 1.14)	0.55	12
Lymphoceles	RCT [30,61] (1235)	12	0.61 (0.34, 1.07)	0.09	10
Malignancies	RCT [23,29,61] (1459)	12	1.16 (0.40, 3.38)	0.79	0
Withdraw	RCT [23,24,27,28,29,30,32,61] (2384)	12	0.98 (0.34, 2.81)	0.97	82 *

^a^ Results reaching statistical significance are in bold font. ^b^ Relatives risk values of <1 favor treatment with TAC. ^c^ *p*: *p*-value for relative risk estimation; I^2^: test for heterogeneity. * The high heterogeneity (*p* < 0.00001) could be caused by the following trials: Mayer 1997 [23], Hardinger 2005 [29] and Vicenti 1996 [32]. Sensitivity analysis showed much reduced heterogeneity (*p* = 0.23, I2 = 29%) when these trials were removed from the analysis.

* The study of Ekberg is a RCT with 12 months of experimental data and 36 months of observational data, so that the article containing results at 36 months results was included in cohort analysis.

#### 3.2.2. AMETAB *vs.* AMETAB

Five articles from four RCTs with 12 months of follow-up compared AZA (control) with MMF (intervention) [49,50,51,52,53]. The dosage used in the studies ranged from 100–150 mg/day for AZA and 2–3 g/day for MMF.

All five articles were included in meta-analysis and the results are shown in Table 3. MMF was associated with an increased risk for total infections and gastrointestinal discomfort, including abdominal pain, diarrhea and vomiting. The sensitivity analysis for heterogeneity indicated that participants who were taking MMF had a higher risk of withdrawing from the treatment.

**Table 3:** Meta-analysis results of outcomes reported by studies comparing MMF *vs.* AZA ^a^.

Outcome	Study Design (N)	Time in months	Relative Risk ^b^	Statistics ^c^	
			(95% CI)	*p*	I^2^
Total Infections	RCT [49,51,52,53] (919)	12	1.17 (1.03, 1.33)	0.01	0
CMV	RCT [49,51,52,53] (919)	12	0.94 (0.82, 1.03)	0.17	41
Abdominal pain	RCT [49,50,53] (873)	12	1.40 (1.06, 1.83)	0.02	14
Diarrhea	RCT [49,50,53] (873)	12	1.49 (1.17, 1.90)	0.001	10
Nausea	RCT [49,50,53] (873)	12	0.98 (0.69, 1.39)	0.91	41
Vomiting	RCT [49,50,53] (873)	12	1.54 (1.10, 2.15)	0.01	0
Malignancies	RCT [49,53] (652)	12	1.52 (0.81, 2.82)	0.19	0
Withdraw	RCT [49,50,53] (873)	12	1.21 (0.77, 1.92)	0.40	66*

^a^ Results reaching statistical significance are in bold font. ^b^ Relatives risk values of <1 favor treatment with MMF. ^c^ *p*: *p*-value for relative risk estimation;I^2^: test for heterogeneity. * RCT Sadek 2002 [53] is largely responsible for the heterogeneity among RCTs that reported withdraw. Sensitivity analysis showed a relative risk of 1.93 (1.06 to 3.52), and dramatically reduced heterogeneity (*p* =0.95, I^2^ = 0%) when this trial was removed from the analysis.

#### 3.2.3. TOR-I *vs.* CNI

Eight articles from five RCTs with 12 months of follow-up and one RCT of 24 months were included in this group: two accessing SRL *vs.* TAC [47,65], four accessing SRL *vs.* CsA [43,44,45,46] and two accessing both [61,62]. In these studies, SRL was used at low (4–8 ng/mL) and standard (10–20ng/mL) doses, as well as CsA, whereas TAC was administered only at low dose. SRL was applied as experimental drug and CsA and TAC served as controls.

Five articles were included in the meta-analysis (Table 4). When compared with any of the CNIs, the use of SRL presented a higher risk for anemia, dyslipidemia, lymphoceles and withdrawal. The association between SRL and anemia was also reported by one cohort of 36 months, which was not included in the pooled analysis, compared with CsA (n = 476; RR = 1.30; 95% CI: 1.05, 1.60; *p* = 0.02) and TAC (n = 472; RR = 1.29; 95% CI: 1.05, 1.60; *p* = 0.02) [62]. A similar result was observed for dyslipidemia when SRL was compared with TAC (n = 472; RR = 1.42; 95% CI: 1.15, 1.76; *p* = 0.001) [62]. Single analysis of a RCT comparing SRL and TAC at 24 months showed similar results with the SRL *vs.* TAC meta-analysis: SRL was associated with increased risk of anemia, dyslipidemia, lymphoceles and withdrawal, and had no difference for malignancy, infections or hypertension [65]. Moreover, the risk of diabetes was higher for SRL, and the risk of CMV infection was higher for CsA.

**Table 4:** Meta-analysis results of outcomes reported by studies comparing SRL *vs.* CsA or TAC ^a^.

	SRL *vs*. CsA					SRL *vs*. TAC				
Outcome	Study Design (N)	Time (mo.)	Relative Risk ^b^ (95% CI)	Statistics ^c^		Study Design (N)	Time (mo.)	Relative Risk ^b^ (95% CI)	Statistics ^c^	
				*p*	I^2^%				*p*	I^2^%
Total Infections	RCT [46,61] (927)	12	0.98 (0.82, 1.18)	0.86	33	−	−	−	−	−
CMV	RCT [43,44,46,61] (1,155)	12	0.46 (0.25, 0.85)	0.01	53 ^d^	RCT [47,61] (924)	12	0.26 (0.03,2.30)	0.23	79
UTI	RCT [43,44,46,61] (1,155)	12	1.04 (0.79, 1.37)	0.79	35	−	−	−	−	−
Anemia	RCT [43,46,61] (1,010)	12	1.48 (1.16, 1.90)	<0.01	0	RCT [47,61] (924)	12	1.56 (1.26,1.93)	<0.01	0
Leukopenia	RCT [43,46,61] (1,010)	12	1.32 (0.70, 2.47)	0.39	57 ^e^	RCT [47,61] (924)	12	0.82 (0.59,1.14)	0.24	0
Dyslipidemia	RCT [43,46,61] (1,010)	12	2.02 (1.03, 3.97)	0.04	65 ^f^	RCT [47,61] (924)	12	1.58 (1.10,2.26)	0.01	0
Diabetes	RCT [43,44,46,61] (1,155)	12	1.82 (1.14, 2.89)	0.05	0	RCT [47,61] (924)	12	0.78 (0.52,1.17)	0.23	0
Hypertension	RCT [43,46,61] (1,010)	12	0.94 (0.66, 1.33)	0.71	28	RCT [47,61] (924)	12	1.53 (0.55,4.23)	0.41	93
Lymphoceles	RCT [44,46,61] (1,072)	12	1.65 (1.10, 2.46)	0.01	18	RCT [47,61] (924)	12	2.92 (1.73,4.93)	<0.01	0
Malignancies	RCT [43,61] (871)	12	1.09 (0.09,13.46)	0.95	60	−	−	−	−	−
Withdraw	RCT [43,44,46,61] (1,155)	12	3.68 (2.22, 6.11)	<0.01	0	RCT [47,61] (924)	12	4.31 (2.32,7.99)	<0.01	0

Abbreviations: UTI, urinary tract infection. ^a^ Results reaching statistical significance are in bold font. ^b^ Relatives risk values of <1 favor treatment with SRL. ^c^ *p*: *p*-value for relative risk estimation; I^2^: test for heterogeneity. ^d^ Sensitivity analysis removing Groth 1998 [43]: RR 0.38 (0.21 to 0.67; I^2^ = 38%). ^e^ Sensitivity analysis removing Groth 1998 [43]: RR 1.0 (0.69 to 1.47; I^2^ = 0%). ^f^ Sensitivity analysis removing Ekberg 2007 [61]: RR 3.01 (1.61 to 5.62; I^2^ = 0%).

Lebranchu *et al.* (2012) reported the 5-years results of the RCT from Buchler *et al.* [44], which compared SRL *vs.* CsA, and reported outcomes as mouth ulcers, acne, UTI, malignancies, diabetes and withdrawal due to adverse event, but none showed difference between groups in single analysis [45].

One RCT with 12 months of follow-up compared SRL in combination with CsA *vs.* SRL alone [64] and reported anemia, hypertension, CMV infection, lymphoceles, dyslipidemia, diabetes and polycythemia; however, only polycythemia was associated with the combination of TOR-I and CNI (n = 207; RR = 3.40; 95% CI: 1.16, 9.98; *p* = 0.03).

#### 3.2.4. CNI + AMETAB *vs.* CNI

Seven studies were included in this group: one cohort with 240 months of follow-up comparing CsA+AZA *vs.* CsA [69], one RCT with 12 months of follow-up comparing CsA + MMF *vs.* CsA [33], three RCTs with 12 and 36 months of follow-up comparing TAC + AZA *vs.* TAC [36,37,38], one RCT with 12 months of follow-up comparing TAC + MMF *vs.* TAC [39] and one RCT with 12 months of follow-up comparing CsA + AZA *vs.* TAC [34]. CsA and MMF were administrated at both low and standard doses, whereas TAC and AZA were used only at the standard dose.

Two studies comparing TAC + AZA *vs*. TAC at 12 months of follow-up were meta-analyzed and the results are displayed on Table 5. The meta-analysis revealed that the combination of TAC and AZA was associated with a greater risk of leukopenia and withdrawal compared with TAC alone. The RCT with 36 months of follow-up confirmed this result for leukopenia (n = 475; RR = 5.60; 95% CI: 2.39, 13.08; *p* < 0.01) [37]. In a single analysis, the combination of TAC and AZA was associated with anemia at 12 months of follow-up (n = 475; RR = 1.55; 95% CI: 1.06, 2.28; *p* = 0.02) [36].

**Table 5:** Meta-analysis results of outcomes reported by studies comparing TAC + AZA *vs.* TAC ^a^.

Outcome	Study Design (N)	Time in months	Relative Risk ^b^ (95% CI)	Statistics ^c^	
				*p*	I^2^
Total Infections	RCT [36,38] (720)	12	0.99 (0.82, 1.20)	0.94	0
Leukopenia	RCT [36,38] (720)	12	8.41 (3.36, 21.02)	<0.01	0
Diabetes mellitus	RCT [36,38] (720)	12	0.85 (0.41, 1.76)	0.67	0
Hypertension	RCT [36,38] (720)	12	0.83 (0.65, 1.06)	0.13	0
Tremor	RCT [36,38] (720)	12	0.96 (0.68, 1.35)	0.82	0
Withdraw	RCT [36,38] (720)	12	10.39 (4.40, 24.56)	<0.01	0

^a^ Results reaching statistical significance are in bold font. ^b^ Relatives risk values of <1 favor treatment with Antiproliferative Agent + TAC. ^c^ *p*: *p*-value for relative risk estimation; I^2^: test for heterogeneity

The combination of TAC and MMF at 12 months was associated with a greater risk of gastritis (n = 135; RR = 1.92; 95% CI: 1.18, 3.14; *p* = 0.009) and leukopenia (n = 153; RR = 3.00; 95% CI: 1.13, 8.01; *p* = 0.03) [39], compared with TAC alone.

The combination of CsA and AZA at 240 months of follow-up was associated with higher risk for hypertension (n = 314; RR = 1.23; 95% CI: 1.04, 1.46; *p* = 0.02) and a lower risk for bacterial infections (n = 314; RR = 0.31; 95% CI: 0.13, 0.76; *p* = 0.01), compared to CsA alone [69]. The studies comparing CsA + MMF *vs*. CsA and TAC + MMF *vs*. TAC reported CMV infection, diabetes, diarrhea and leukopenia but the estimated RRs were not significant.

#### 3.2.5. TOR-I *vs.* AMETAB

Seven RCTs were included in this group: four comparing EVL *vs*. MMF, at 12 months [54,59], 24 months [60], and 36 months of follow-up [55,56] and three comparing SRL *vs*. MMF at 12 months [57,58] and 24 months of follow-up [63]. SRL and MMF were used at standard doses, and EVL was used at low (1.5 mg/day) and high (3 mg/day) doses.

The studies comparing EVL and MMF were meta-analyzed in subgroups of 12 and 36 months of follow-up and divided based on low- and high-dose EVL (Table 6). Additionally, the studies comparing SRL and MMF at 12 months of follow-up were meta-analyzed. No differences were observed when comparing low- and high-dose EVL. Independent of dosing, EVL was associated with an increased risk of dyslipidemia and withdrawal. MMF presented a higher risk of CMV infection when compared with both doses of EVL. The single analysis for 24 months of follow-up showed similar results: both doses of EVL were associated with increased risk of withdrawal and lower risk of CMV infection and leukopenia when compared with MMF [60]. In the same study, high-dose EVL was associated with a greater risk of diabetes (n = 833; RR = 1.96; 95% CI: 1.18, 1.87; *p* = 0.01) [60]. Compared with MMF, SRL showed increased risk of withdrawal in meta-analysis and no significant results were found in single-analysis.

#### 3.2.6. AMETAB *vs.* CNI

Three studies compared an antimetabolite with a CNI: one RCT with 63 months of follow-up that compared MMF *vs.* TAC [39] and two studies comparing AZA *vs.* CsA, a cohort with 240 months of follow-up [69] and a RCT with 36 months of follow-up [40]. All drugs were used at standard doses.

There was not a sufficient number of studies with identical follow-up periods to perform meta-analysis. The risk for dyslipidemia (n = 132; RR = 1.75; 95% CI: 1.13, 2.71; *p* = 0.01) and diarrhea (n = 132; RR = 3.87; 95% CI: 1.35, 11.03; *p* = 0.01) was higher for MMF, compared with TAC, and MMF presented a lower risk of withdrawal than TAC (n = 132; RR = 0.14; 95% CI: 0.04, 0.45; *p* = 0.0009) [39].

AZA was associated with an increased risk of pulmonary infections (n = 276; RR = 2.25; 95% CI: 1.01, 5.00; *p* = 0.05) and leukopenia (n = 276; RR = 2.76; 95% CI: 1.86, 4.08; *p* < 0.01) [40] and a lower risk of hypertension (n = 205; RR = 0.77; 95% CI: 0.61, 0.97; *p* = 0.03) [69].

## 4. Conclusions

Six different groups of immunosuppressant drugs were evaluated and compared. Evaluating the safety of immunosuppressive drugs is complex because kidney transplantation requires the simultaneous use of multiple classes of drugs at varying doses.

The majority of the studies included here showed a low risk of bias, and only one study revealed a high risk of bias for allocation order generation and allocation confidentiality [34]. Based on the parameters described in the Cochrane Handbook [19], the quality of most studies was compromised by a lack of sufficient information to judge the randomization and allocation concealment.

However, this quality assessment did not invalidate the results of the meta-analysis. Overall, the heterogeneity of the treatment-efficacy results was low, indicating small inter-study variability. In general, the observational studies did not show selection bias, and the majority of these studies used the same time in and out of treatment, allowing for a comparison of populations.

**Table 6:** Meta-analysis results of outcomes reported by studies comparing EVL or SRL *vs.* MMF ^a^.

Outcome	EVL (ld) *vs.* MMF					EVL (hd) *vs*. MMF					SRL *vs*. MMF				
	Study Design (N)	Time (mo.)	Relative Risk ^b^ (95% CI)	Statistics ^c^		Study Design (N)	Time (mo.)	Relative Risk ^b^ (95% CI)	Statistics ^c^		Study Design (N)	Time (mo.)	Relative Risk ^b^ (95% CI)	Statistics ^c^	
				*p*	I^2^				*p*	I^2^				*p*	I^2^
**Total Infections**	RCT [54,59] (946)	12	0.62 (0.26, 1.48)	0.28	92	RCT [54,59] (946)	12	0.83 (0.58, 1.18)	0.29	70	−	−	−	−	−
**CMV infections**	RCT [54,59] (946)	12	0.23 (0.12, 0.42)	<0.01	0	RCT [54,59] (946)	12	0.15 (0.01, 2.17)	0.16	73	−	−	−	−	−
	RCT [55,56] (781)	36	0.47 (0.16, 1.41)	0.18	78	RCT [55,56] (780)	36	0.47 (0.29, 0.74)	<0.01	0					
**Anemia**	RCT [54,59] (946)	12	0.97 (0.79, 1.20)	0.80	0	RCT [54,59] (946)	12	1.15 (0.95, 1.40)	0.15	0	−	−	−	−	−
	RCT [55,56] (781)	36	1.17 (0.73, 1.88)	0.50	76	RCT [55,56] (780)	36	1.47 (0.97, 2.23)	0.07	74					
**Leukopenia**	RCT [55,56] (781)	36	0.50 (0.24, 1.06)	0.07	42	−	−	−	−	−	−	−	−	−	−
**Dyslipidemia**	RCT [54,59] (946)	12	1.68 (1.01, 2.79)	0.05	68	RCT [54,59] (946)	12	1.63 (1.08, 2.46)	0.02	52	−	−	−	−	−
**Hypertension**	RCT [54,59] (946)	12	0.98 (0.73, 1.32)	0.87	0	RCT [54,59] (946)	12	0.97 (0.80, 1.18)	0.78	0	−	−	−	−	−
**Lymphoceles**	RCT [55,56] (781)	36	1.54 (0.96, 2.45)	0.07	14	RCT [55,56] (780)	36	2.08 (1.00, 4.32)	0.05	63	−	−	−	−	−
**Withdraw**	RCT [55,56] (781)	36	1.23 (1.07, 1.43)	0.005	0	RCT [55,56] (780)	36	1.41 (1.23, 1.62)	<0.01	0	RCT [57,58] (459)	12	1.81 (1.20, 2.72)	0.004	0

Abbreviations: (ld), Low dose; (hd), High dose; mo., months. ^a^ Results reaching statistical significance are in bold font. ^b^ Relatives risk values of <1 favor treatment with TOR-I. ^c^ *p*: *p*-value for relative risk estimation; I^2^: test for heterogeneity.

Compared with CsA, treating kidney transplant patients with TAC resulted in a higher risk for diabetes, whereas those taking CsA had a greater risk of developing dyslipidemia. A retrospective study of risk factors for new-onset diabetes after transplantation (NODAT) found that higher tacrolimus concentrations were an independent predictor of NODAT [70]. In a meta-analysis comparing TAC *vs.* CsA as the primary immunosuppressant for kidney transplant recipients, TAC-treated patients were two to three times more likely to develop new diabetes mellitus that required insulin. However, the adverse events associated to CsA (constipation, hirsutism, and gingival hyperplasia) were different from those that we found, likely due to the time of use [16].

In regard to MMF *vs.* AZA, the majority of studies reported a larger number of adverse events for the groups treated with MMF at 12 months of follow-up. The meta-analyses of total infections, vomiting, diarrhea, and abdominal pains statistically favored treatment with AZA. The results of the present meta-analysis agree with the findings of a systematic review conducted in 2009 [71], which found that MMF-treated patients had a greater risk of diarrhea, whereas the risks of CMV infection, anemia, leukopenia and malignancy were not significant.

Our results showed that the use of SRL was associated with higher risk for anemia, dyslipidemia, lymphoceles and withdrawal compared with any CNI. There were no significant differences for infections, UTI, leukopenia, hypertension, or malignancies. These results agree with the findings of a multicenter study which used TAC in combination with different doses of SRL and showed that the incidence of dyslipidemia (hypercholesterolemia) was associated with higher doses of SRL [72]. Another study comparing TOR-I versus CNI found an increased risk of bone marrow suppression outcomes (leukopenia, thrombocytopenia, and anemia), lymphoceles and dyslipidemia for patients taking SRL [73]. Compared with CsA, SRL presented a higher risk for diabetes and reduced risk of CMV infection. Although regimens containing SRL have a higher risk of post-transplant diabetes than regimens without SRL [74], TAC has a higher risk for diabetes than CsA, thus the difference of risk between TAC and SRL may have no significance. Johnston *et al.* compared SRL with TAC and with CsA and found that patients treated with CsA had the lowest incidence of diabetes (15.6%), followed by SRL (17.8%) and then TAC (19%) [74]. Sirolimus in combination with CNI may increase clinically significant adverse events, such as CNI-related nephrotoxicity and dyslipidemia. Other outcomes include hematologic side effects and a higher incidence of lymphoceles [75]. Furthermore, the use of SRL combined with TAC might increase the risk of post-transplant diabetes mellitus [17,76].

As the majority of studies comparing TOR-I with CNI assess CNI minimization or elimination thru conversion from CNI to TOR-I, the number of studies with such comparison included in the present review was limited, once conversion of drugs was considered exclusion criteria.

Independent of the dose, EVL was associated with increased risk of dyslipidemia and withdrawal. MMF presented higher risk of CMV infection compared with both doses of EVL, but there was no difference in bone marrow suppression (leukopenia and anemia), hypertension, lymphoceles and infections. According to one study, SRL and MMF are associated with similar incidences of both leukopenia and thrombocytopenia [77]. This study reported similar incidences of leukopenia with the combination of MMF and SRL and MMF alone, and similar incidences of thrombocytopenia were observed between their combination and SRL alone, indicating no difference in the risk of these outcomes [77]. The definitions of diabetes and other diseases, such malignancies and dyslipidemia, vary a lot between studies, thus the interpretation of results regarding these diseases should consider the differences between definitions. Many of the clinical trials included were funded by pharmaceutical industries, limiting the interpretation of results, as these companies may benefit from reporting only favorable findings.

Current immunosuppressive protocols use combinations of immunosuppressive agents with different mechanisms of action to maximize efficacy and minimize the toxicity of each drug. The appearance of new immunosuppressive agents and tolerance protocols emerge shows potential as a means to deliver immunosuppression without long-term toxicity. In this regard, belatacept is a second-generation costimulation blocker that in phase 3 trials was to provide effective immunosuppression while avoiding the toxicities associated with calcineurin inhibitors [78].

Modifications are still being introduced in immunosuppressant protocols to take advantage of the drugs’ beneficial actions and to reduce the adverse events. Although safety information alone is not enough to base decision making in health, together with reliable information about the long-term efficacy of immunosuppressants, the results of the present review might assist healthcare professionals and managers in choosing the best immunosuppressant regimen. We concluded that the data examined in this meta-analysis are similar to those describe by others authors. Adverse reactions were observed in all classes of immunosuppressive drugs; thus the choice of treatment must be made by the clinical staff based on specific patient characteristics.
